# Cultural Humility and Recovery-Promoting Competencies Among Mental Health Practitioners: The Mediating Role of Recovery Attitudes

**DOI:** 10.1007/s10488-026-01513-x

**Published:** 2026-06-17

**Authors:** Subin Na, Suzanne S. Tham, Phyllis Solomon

**Affiliations:** School of Social Policy & Practice, University of Pennsylvania, Pennsylvania, Philadelphia USA

**Keywords:** Cultural humility, Recovery-promoting competency, Recovery attitudes, Mental health practitioners, Serious mental illness

## Abstract

Recovery-oriented mental health practice represents a paradigm shift toward holistic, client-centered care prioritizing autonomy, hope, and social integration for individuals with serious mental illness. Mental health practitioners are instrumental in facilitating recovery; however, mechanisms through which practitioner orientations translate into recovery-promoting competencies remain underexamined. Individuals with serious mental illness face disparities arising from stigma and structural inequities. Cultural humility, characterized by ongoing self-reflection, power awareness, and commitment to equitable partnership, has emerged as a critical orientation to operationalize recovery values. Yet mechanisms linking cultural humility to recovery-promoting competencies remain unclear. This study examines whether recovery attitudes mediate the relationship between cultural humility and recovery-promoting competencies among mental health practitioners. Data were collected via anonymous survey from 188 mental health practitioners (June 2024–March 2025) employed in U.S. mental health organizations providing clinical services to adults with serious mental illness. Structural equation modeling tested a mediation model using three latent variables: cultural humility, recovery attitudes, and recovery-promoting competencies. Results showed cultural humility was significantly associated with recovery attitudes (β = 0.638, *p* < .001), which in turn was associated with recovery-promoting competencies (β = 0.654, *p* < .001). Recovery attitudes mediated 54.9% of the total effect of cultural humility on recovery-promoting competencies (indirect effect β = 0.417, 95% CI [0.310, 0.670], *p* < .001). This relationship remained robust after controlling for demographic and professional covariates. Findings provide empirical evidence that cultural humility operates through recovery attitudes to shape practitioner behavior, underscoring the importance of cultivating cultural humility within supervisory and organizational contexts.

## Introduction

Recovery is the prevailing framework in mental health services (Gyamfi et al., 2025; Pincus et al., 2016), defined as the real-life experiences of individuals leading satisfying lives despite limitations caused by serious mental illness (SMI) (Anthony, 1993; Deegan, 1988). Recovery-oriented approaches prioritize individuals’ well-being, autonomy, and resilience, representing a fundamental shift from traditional medical models toward a holistic approach that emphasizes hope, personal agency, and community integration (Carpenter, 2002; Mancini, 2008; SAMHSA, 2012). Within this paradigm, mental health practitioners play an instrumental role in facilitating recovery, exerting influence that is both profound and complex. Practitioners may function as catalysts for empowerment or, conversely, as barriers to recovery (Deegan, 2000; Russinova et al., 2013). The quality of client–practitioner interactions significantly shapes recovery trajectories, yet remains fragile and highly contingent on practitioners’ approaches. Deegan (2000) characterized negative clinical interactions that leave clients feeling dehumanized, undervalued, and disempowered as having a “spirit-breaking” effect. In contrast, practitioners can positively influence recovery through strong therapeutic alliances and empowering clinical practices that acknowledge clients’ individuality, foster hope and empowerment, and support illness management (Deegan, 2000; Howgego et al., 2003; Minkoff, 1987; Russinova et al., 2013).

Central to understanding this positive influence is recognizing the relational nature of recovery. The CHIME (Connectedness, Hope/Optimism, Identity, Meaning in Life, and Empowerment) framework identifies connectedness, defined as relationships and support from others, as foundational elements of recovery (Leamy et al., 2011). For individuals with SMI, practitioners often constitute an important source of connectedness within their recovery journey. This therapeutic connectedness is grounded in practitioner attributes consumers and practitioners identify as central to recovery-oriented care, including respect, trust, accessibility, belief in clients’ potential, and demonstrated support for coping (Williams & Tufford, 2012). Building on these relational foundations, Na et al. (2025) identified six interconnected practitioner-level competencies that operationalize recovery-oriented practice: person-centered care, empowerment, holistic well-being, self-reflexive and inclusive attitudes, social justice advocacy, and meaningful occupation support. Collectively, these competencies constitute recovery-promoting competencies, defined as a complex set of practitioner capabilities encompassing knowledge, skills, abilities, and personal characteristics that facilitate recovery processes (Marrelli et al., 2005; Russinova et al., 2013).

Empirical research demonstrates the significance of recovery-promoting competencies in positively influencing personal recovery outcomes (Jas & Wieling, 2018; Moran et al., 2014; Sánchez-Guarnido et al., 2024) as well as strengthening the working alliance between clients and providers (Moran et al., 2014). Despite this evidence, translating recovery philosophy into consistent and competent practice remains an ongoing implementation challenge (Le Boutillier et al., 2011). Accordingly, understanding what drives variation in practitioners’ recovery-promoting competencies and identifying mechanisms through which these competencies develop remains a critical area for empirical investigation.

### Cultural Humility in Recovery-Oriented Practice

Individuals with SMI face compounded disparities arising from stigma and intersecting structural inequities across multiple marginalized identities. These interlocking systems shape service access, care quality, and recovery trajectories, while public stigma and discrimination limit social inclusion, employment, and housing opportunities, ultimately hindering recovery (Tham & Solomon, 2025; Weerasinghe et al., 2023). Building on this, stigma toward individuals with SMI is not confined to the general public. Corrigan and Nieweglowski (2019) proposed that although greater familiarity with mental illness typically reduces public stigma, this inverse relationship does not consistently hold for mental health service providers, whose professional familiarity with SMI can paradoxically coexist with stigmatizing attitudes. Empirical syntheses indicate that provider stigma often manifests as therapeutic pessimism regarding the likelihood of recovery, a stance that itself functions as a barrier to recovery-oriented engagement and is experienced by service users as stigmatizing (Knaak et al., 2017). Of particular relevance for workforce development, training itself appears implicated in shaping and perpetuating these attitudes. In a review of factors contributing to psychology’s underrepresentation in SMI care, O’Connor and Yanos (2021) identified clinician stigma alongside limited SMI-specific training as key perpetuating factors, and a subsequent empirical study found that both program-level training factors and individual characteristics predicted clinical psychology doctoral students’ attitudes toward SMI (O’Connor & Yanos, 2023). These findings indicate that clinical preparation can either mitigate or inadvertently reinforce stigma, and they highlight the need to identify practitioner orientations, such as cultural humility, that may counteract stigmatizing tendencies and promote recovery-oriented engagement, having implications for how practitioners are trained, supervised, and supported within mental health organizations. Against this backdrop of stigma in both public and provider contexts, cultural humility has emerged as a critical orientation in mental health practice as policy efforts have increasingly emphasized culturally responsive care. For example, the U.S. Department of Health and Human Services Office of Minority Health released the National Standards for Culturally and Linguistically Appropriate Services (CLAS), calling for equitable and respectful care responsive to diverse cultural and communication needs (Office of Minority Health, 2013). However, despite this policy attention, challenges in implementation persist (Aggarwal et al., 2017; Barksdale et al., 2014; Tham & Solomon, 2024).

To address these implementation gaps, cultural humility offers a process-oriented complement to cultural competence. While cultural competence has long guided multicultural training, accumulating evidence suggests it has limited impact on service user outcomes (Beach et al., 2005; Truong et al., 2014) and risks treating culture as a discrete body of knowledge to be mastered, potentially reinforcing stereotypes (Lekas et al., 2020; Tham & Solomon, 2024). These limitations are particularly consequential in SMI care, where mental illness stigma routinely intersects with marginalization based on race, gender, sexuality, and socioeconomic position (Holley et al., 2016), and where the goal is not merely culturally informed treatment but the cultivation of equitable, partnership-based relationships in which service users are recognized as experts in their own recovery. Tervalon and Murray-García (1998) defined cultural humility as incorporating ‘a lifelong commitment to self-evaluation and critique, to redressing the power imbalances in the physician–patient dynamic, and to developing mutually beneficial and non-paternalistic partnerships with communities on behalf of individuals and defined populations’ (p. 123). Whereas cultural competence is content-driven with an implied endpoint, cultural humility is process-oriented and ongoing, foregrounding self-reflection, awareness of power and privilege, and recognition of service users as experts in their own lives (Tham & Solomon, 2024). The two are complementary rather than mutually exclusive (Tham & Solomon, 2024), but cultural humility’s emphasis on relational practice and partnership aligns more directly with the foundations of recovery-promoting practice than does cultural competence alone. Consistent with this framing, Foronda et al. (2016) identified five core principles: openness, self-awareness, egolessness, supportive interactions, and continuous self-reflection.

Cultural humility and recovery-oriented mental health practice intersect in meaningful ways (Tham & Solomon, 2024, 2025). Both frameworks recognize individuals as experts in their own recovery journey and emphasize collaborative therapeutic relationships with those in recovery. Cultural humility requires practitioners to engage in ongoing self-reflection and critique, addressing power imbalances and building interpersonal sensitivity toward service users’ cultural identities and lived experiences (Tham & Solomon, 2025). Recovery-oriented practices similarly prioritize self-determination, partnership, and the valuing of service users’ expertise (Na et al., 2025). When providers adopt cultural humility into their interactions with service users, it encompasses underlying principles and values consistent with recovery-oriented practices, including dignity and respect, collaboration and partnership, person-centered care, and support for self-determination and autonomy (Tham & Solomon, 2024). Accordingly, higher levels of practitioner cultural humility may enhance providers’ capacity to engage in recovery-promoting practice with individuals with SMI.

Tham and Solomon (2025) also recently proposed a comprehensive framework operationalizing culturally safe recovery-oriented practice across four interconnected domains: ways of knowing (critical reflexivity beyond cultural competence), ways of presence (self-reflection and power awareness), ways of doing (tangible actions dismantling power imbalances), and ways of operating (organizational accountability). This framework illustrates that recovery is deeply embedded in socio-cultural contexts and requires addressing both intrapersonal and structural dimensions. Despite this conceptual and theoretical alignment, the practical mechanisms linking cultural humility to recovery-promoting competencies remain unclear. Building on Tham and Solomon’s (2024, 2025) framework that clarifies what culturally safe recovery practice looks like holistically, understanding the specific processes through which practitioners’ reflective, power-aware orientations translate into concrete, competent practice behaviors represents an important empirical gap.

### Recovery Attitudes as a Mechanism Linking Cultural Humility to Practice Competencies

Recovery attitudes represent practitioners’ endorsement of personal recovery principles, operationalized as belief that recovery is possible and multidimensional, valuation of service users’ autonomy and self-determination, and commitment to supporting life goals beyond symptom management (Badu et al., 2025; Borkin et al., 2000; Gyamfi et al., 2020). These attitudes play a critical role in shaping recovery-supportive environments and translating recovery values into practice (Bedregal et al., 2006; Chang et al., 2025; Mak et al., 2018; Salyers et al., 2013). While prior research has identified recovery attitudes as important to practice, the mechanisms through which recovery attitudes influence observable competencies and the extent to which they interact with other practitioner-level orientations such as cultural humility remain empirically underexamined.

Recovery attitudes may function as a key mechanism linking cultural humility to recovery-promoting competencies among mental health practitioners. According to the Theory of Reasoned Action (TRA), behavioral intentions are determined by attitudes toward the behavior, which are themselves constructed through individuals’ evaluation of behavioral outcomes weighted by the importance they assign to those outcomes (Fishbein & Ajzen, 1975). Applied to recovery-oriented practice, this framework suggests that practitioners’ behavioral beliefs about the value and importance of cultural humility shape recovery-oriented attitudes, which in turn drive recovery-promoting competency. Specifically, cultural humility represents the foundational layer of behavioral beliefs and their evaluations. Cultural humility is characterized by ongoing self-reflection, openness to learning, and critical awareness of power differentials and positionality within therapeutic relationships and broader organizational systems (Curtis et al., 2019; Tham & Solomon, 2025). Through this reflective process, practitioners critically examine dominant assumptions about mental illness and their own roles as professionals, developing evaluated beliefs about the significance of centering service users’ perspectives, addressing power imbalances, and building equitable partnerships.

Recovery attitudes represent the attitude component of TRA, reflecting practitioners’ overall favorable or unfavorable orientation toward recovery-oriented principles. These attitudes encompass faith that recovery is possible despite symptoms, recognition that recovery trajectories differ, valuation of service user agency and self-determination, and acknowledgment that while stigma may impede recovery, it does not prevent it (Borkin et al., 2000). Within the TRA framework, recovery attitudes are not independent of cultural humility; rather, they emerge from the behavioral beliefs and evaluations that characterize cultural humility. Through ongoing self-reflection and critical examination of power dynamics, practitioners develop robust recovery attitudes grounded in genuine commitment to service user autonomy and belief in recovery’s possibility.

These recovery attitudes, in turn, function as the motivational mechanism that translates practitioners’ reflective and power-aware orientations into behavioral intention to perform recovery-promoting behaviors. When practitioners hold well-developed, positive recovery attitudes grounded in cultural humility’s evaluative stance, they are more likely to intend to engage service users as active, empowered participants in the therapeutic relationship. This behavioral intention is reflected in practitioners’ perceived recovery-promoting competencies across multiple domains, including fostering hope and self-confidence, supporting self-determination and autonomy, demonstrating genuine understanding and non-judgmental acceptance, building trusting relationships, and advocating for clients’ needs (Russinova et al., 2013). In this way, the TRA framework clarifies a specific pathway: cultural humility, as a system of behavioral beliefs and evaluations regarding power, partnership, and service user expertise, shapes the recovery attitudes that motivate practitioners’ intention to perform recovery-promoting competencies in clinical practice.

### Current Study and Research Questions

Despite increasing emphasis on cultural humility and recovery-oriented care within mental health systems, little empirical research has examined the extent to which cultural humility translates into recovery-promoting practice among practitioners. In particular, recovery attitudes have rarely been empirically tested as a mechanism linking cultural humility to recovery-promoting competencies. Addressing this gap may clarify practitioner-level pathways through which recovery-oriented practices are enacted in routine clinical practice.

Accordingly, this study examines whether recovery attitudes mediate the relationship between cultural humility and recovery-promoting competencies among mental health practitioners working with individuals with SMI. Two research questions guide the analysis: (1) To what extent is cultural humility associated with recovery-promoting competencies among mental health practitioners? (2) Do recovery attitudes mediate the relationship between cultural humility and recovery-promoting competencies? Based on the literature reviewed, we hypothesize that practitioners with higher levels of cultural humility will demonstrate greater recovery-promoting competencies and that recovery attitudes will mediate the association between cultural humility and recovery-promoting competencies.

## Methods

### Sample and Data Collection

This study employed a cross-sectional design, with data collected through an anonymous Qualtrics survey administered between June 2024 and March 2025. The final sample included 207 mental health practitioners employed in U.S. mental health organizations (excluding private practice) who were actively providing clinical services to adults with SMI. Eligibility criteria required that at least 40% of a practitioner’s caseload consist of clients diagnosed with SMI. Participants represented diverse professional roles and service settings, including inpatient units, outpatient programs, and residential treatment facilities.

Several procedures were implemented to ensure the integrity of the analytic sample. Qualtrics fraud-prevention tools (Bot Detection, RelevantID, Security Scan Monitor, and IP-based duplicate checks) were activated to minimize the risk of automated or invalid responses. In addition, geolocation metadata were examined to identify and exclude responses originating from non-US IP addresses, given that the study population was U.S.-based mental health practitioners; nine such responses were excluded. To identify careless or insufficient effort responding, long-string analyses were conducted, which is a commonly used and intuitive detection strategy (Curran, 2016). This method involves examining the longest sequence of identical responses provided by each participant, based on the assumption that attentive respondents are unlikely to select the same answer for many consecutive items (Curran, 2016). Ten respondents provided more than 20 consecutive identical responses across the 77-item survey and were removed from the dataset, resulting in a final analytic sample of 188 (see Table 1). To further assess response bias, the 13-item short form of the Marlowe–Crowne Social Desirability Scale (Reynolds, 1982) was administered. The sample mean score was 5.16, indicating values within the expected normative range.

### Recruitment

Participants were recruited through a non-probability snowball sampling method. Recruitment efforts included: (1) sharing the survey link through the authors’ professional social media accounts (e.g., LinkedIn) within online networks of mental health practitioners; (2) asking colleagues in related disciplines (such as Master of Social Work alumni and clinical psychology doctoral students) to distribute the invitation across their professional and social circles, including platforms like Bluesky and Twitter; (3) partnering with a nonprofit executive connected to the Philadelphia Mental Health Care Corporation to further circulate the survey among professional contacts; and (4) locating mental health agencies across various U.S. states through publicly available directories and requesting that organizational representatives forward the survey to eligible employees. Participants who completed the survey could opt into a raffle for one of five $100 Amazon gift cards. All study procedures received approval from the Institutional Review Board at the University of Pennsylvania, and electronic informed consent was obtained from all respondents prior to participation.

**Table 1:** Participant Characteristics (*N* = 188)

Characteristics	*n* (%)	Mean (SD; Min – Max)
Age		40.28 (9.26; 22–71)
Years of working experience		10.54 (7.58; 1–46)
Gender		
Female	110 (58.5)	
Male	74 (39.4)	
Non-binary	4 (2.1)	
Education		
Less than high school	1 (0.5)	
High school or GED	3 (1.6)	
Some college	4 (2.1)	
Technical school	2 (1.1)	
Bachelor’s degree	62 (33.0)	
Master’s degree	95 (50.5)	
PhD or higher	21 (11.2)	
Profession		
Counselor	39 (20.7)	
Psychiatric nurse	12 (6.4)	
Psychiatrist	31 (16.5)	
Psychologist	14 (7.4)	
Social worker	55 (29.3)	
Therapist	33 (17.6)	
Other	4 (2.1)	
Race / Ethnicity		
Asian	14 (7.4)	
Black or African American	26 (13.8)	
Hispanic or Latino	13 (6.9)	
Native Hawaiian or Other Pacific Islander	6 (3.2)	
White	120 (63.8)	
Two or More	8 (4.3)	
Prefer not to answer	1 (0.5)	
Facility Type		
Community-based	94 (50.0)	
Hospital-based	77 (41.0)	
Other	17 (9.0)	
Program Type		
Inpatient facility	43 (22.9)	
Outpatient facility	75 (39.9)	
Residential facility	52 (27.7)	
Other	18 (9.6)	
Facility Size		
Less than 15 clients	23 (12.2)	
15–29 clients	50 (26.6)	
30–59 clients	45 (23.9)	
60–119 clients	28 (14.9)	
120 or more clients	42 (22.3)	
Ownership Type		
For-profit	79 (42.0)	
Non-profit	83 (44.1)	
Government	25 (13.3)	
Other	1 (0.5)	
Average Number of Caseloads		29.32 (36.51; 3–250)

### Measures

Recovery-promoting competency, the dependent variable, was assessed using the Recovery-Promoting Competencies Self-Assessment (RPC; Farkas et al., 2016). RPC is a 24-item self-report instrument rated on a 7-point Likert scale. The scale was designed to measure practitioners’ use of recovery-oriented practices across two conceptual domains: (1) core interpersonal skills and (2) recovery-oriented strategy skills. In the present sample, internal consistency was excellent (Cronbach’s α = 0.92).

Cultural humility, the independent variable, was measured using the Cultural Humility Self-Assessment Scale (CHS-A; Whatley et al., 2024). CHS-A includes 9 items rated on a 5-point Likert scale and assesses providers’ self-perceived cultural humility across key dimensions of cultural identity (e.g., gender, race/ethnicity, sexual orientation, and religion/spirituality). Internal consistency in the current study was good (α = 0.85).

Recovery attitudes, mediating variable, were assessed using Recovery Attitudes Questionnaire–7 (RAQ–7; Borkin et al., 2000). RAQ-7 is a 7-item measure with established concurrent validity assesses providers’ beliefs and openness toward consumer recovery. Internal consistency in this sample was good (α = 0.86).

### Statistical Analyses

All analyses were conducted in R (version 4.3.1) using lavaan package (Rosseel, 2012) for structural equation modeling. Missing data were minimal (< 5%) and handled using full information maximum likelihood (FIML) estimation. Exploratory factor analysis (EFA) with promax rotation was initially conducted on 24-item PRC scale to identify underlying factor structure. Items with factor loadings ≥ 0.60 were retained. The same procedure was applied to the CHS-A scale and RAQ–7 scale. Internal consistency of the final item sets was assessed using Cronbach’s alpha.

Confirmatory factor analysis (CFA) was conducted to validate the measurement model. Model fit was evaluated using robust fit indices (Yuan-Bentler correction) including Comparative Fit Index (CFI; ≥ 0.90 indicating adequate fit), Root Mean Square Error of Approximation (RMSEA; ≤ 0.05 indicating good fit, 0.05–0.08 indicating adequate fit), and Standardized Root Mean Square Residual (SRMR; ≤ 0.08). MLR (Maximum Likelihood Robust) estimator was used to account for potential violations of multivariate normality.

A mediation model was specified with three latent variables (Cultural Humility, Recovery Attitudes, and Recovery-Promoting Competencies) and their respective observed indicators. Two models were tested: (a) unadjusted model examining direct and indirect effects, and (b) adjusted model controlling for demographic (age, gender, race, education) and professional (years working, caseload) covariates. Both models were estimated using MLR estimation with FIML. Indirect effects were estimated using nonparametric bootstrapping with 5,000 resamples to obtain 95% bias-corrected confidence intervals. For this step, ML estimator was used to allow for bootstrap resampling, consistent with standard practice in structural equation modeling.

## Results

### Preliminary Analyses and Exploratory Factor Analysis

Internal consistency was strong for all measures (α ≥ 0.85). Bivariate correlations showed significant positive relationships among all three variables (*r* = .55 to 0.78, all *p* < .001), with no multicollinearity concerns. Among continuous covariates, caseload was significantly associated with recovery attitudes (*r* = .21, *p* = .004) and recovery-promoting competencies (*r* = .15, *p* = .039); age and years of working experience were not significantly correlated with any main variable. Among categorical covariates, education differed significantly across cultural humility (*p* = .004) and recovery attitudes (*p* = .037), and gender differed across recovery-promoting competencies (*p* = .026); race showed no significant differences. All structural paths nonetheless remained significant after adjusting for these covariates (see Table 2). EFA was conducted to refine the three study measures. For RPC, a one-factor solution produced consistently strong loadings across the retained items (all loadings ≥ 0.60), accounting for substantial variance. For CHS and RA, similar approaches supported retention of six items each, meeting 0.60 threshold. These results supported the unidimensional representation of each construct with retained items (see Table 3).

**Table 2:** Descriptive Statistics and Bivariate Correlations

Variable	M	SD	1	2	3
1. Cultural Humility	2.00	1.07	-		
2. Recovery Attitudes	2.64	0.97	0.55**	-	
3. Recovery-Promoting Competency	3.84	1.18	0.68**	0.78**	-
*Continuous covariates (Pearson r)*					
4. Age	40.28	9.26	−0.12	0.12	−0.10
5. Years working	10.54	7.58	0.04	0.07	−0.04
6. Caseload	29.32	36.51	0.04	0.21**	0.15*
*Categorical covariates (one-way ANOVA*,* F)*					
7. Gender			1.75	1.73	3.71*
8. Race			1.93	0.60	1.22
9. Education			3.61**	2.43*	2.24

*p < .05, **p < .01, ***p < .001. Correlations among main variables (rows 1–3) and continuous covariates (rows 4–6) are Pearson r values; entries for categorical covariates (rows 7–9) are F statistics from one-way ANOVAs predicting each main variable. Cronbach’s α: CHS = 0.86, RA = 0.86, RPC = 0.94

**Table 3:** Full EFA Loadings for All Scale Items

Scale	Factor Loading
Recovery-Promoting Competency	
RPC1: Help clients recognize their strengths.	0.713
RPC2: Help clients see the glass as “half-full” instead of “half-empty”.	0.495
RPC3: Help clients put things in perspective.	0.587
RPC4: Help clients feel they can have a meaningful life.	0.582
RPC5: Help clients not to feel ashamed about their psychiatric condition.	0.746
RPC6: Help clients recognize their limitations.	0.364
RPC7: Help clients find meaning in living with a psychiatric condition.	0.642
RPC8: Help clients stand up for themselves.	0.552
RPC9: Encourage clients to take chances and try things.	0.618
RPC10: Remind clients of their achievements.	0.649
RPC11: Help clients feel good about themselves.	0.679
RPC12: Help clients learn from challenging experiences.	0.713
RPC13: Help your clients feel hopeful about the future.	0.680
RPC14: Help clients build their self-confidence.	0.632
RPC15: Help clients to develop ways to live with their psychiatric condition.	0.651
RPC16: Help clients understand the nature of their psychiatric condition.	0.669
RPC17: Demonstrate that you trust your clients.	0.657
RPC18: Demonstrate that you accept clients’ down times.	0.705
RPC19: Demonstrate that you understand your clients.	0.698
RPC20: Demonstrate that you really listen to what your clients have to say.	0.649
RPC21: Demonstrate that you care about your clients.	0.646
RPC22: Demonstrate that you treat your clients with respect.	0.676
RPC23: Demonstrate that you see your clients as people and not just a diagnosis.	0.725
RPC24: Demonstrate that you believe in your clients.	0.738
Cultural Humility	
CH1: I want to learn more about people from other cultural backgrounds.	0.723
CH2: Learning about other cultural backgrounds is an important step in communicating effectively.	0.670
CH3: People from different countries and cultures have perspectives that I can benefit from.	0.790
CH4: I enjoy hearing people talk about their culture.	0.290
CH5: I make an effort to honor the beliefs, customs, and values of other cultures.	0.652
CH6: I am fairly open to exploring the backgrounds of people from other countries and cultures.	0.532
CH7: Learning about other cultures enhances a person’s communication skills.	0.617
CH8: It is important to be considerate of cultural norms and backgrounds that are different than my own.	0.548
CH9: I try to keep an open mind about other cultural norms, beliefs, and values.	0.770
Recovery Attitudes	
RA1: To recover requires faith.	0.455
RA2: Recovery can occur even if symptoms of mental illness are present.	0.729
RA3: Recovering from mental illness is possible no matter what you think may cause it.	0.624
RA4: All people with serious mental illness can strive for recovery.	0.692
RA5: People in recovery sometimes have setbacks.	0.740
RA6: Stigma associated with mental illness can slow down the recovery process.	0.677
RA7: People differ in the way recover from a mental illness.	0.763

### Confirmatory Factor Analysis

The measurement model demonstrated adequate fit to the data, with robust fit indices indicating good model fit (CFI = 0.918, RMSEA = 0.056 [90% CI: 0.045, 0.065], SRMR = 0.054). All factor loadings were statistically significant (*p* < .001), ranging from 0.661 to 0.771 for Cultural Humility Scale, 0.604 to 0.760 for Recovery Attitudes, and 0.620 to 0.766 for Recovery-Promoting Competencies. The three latent factors were moderately to highly correlated (correlations ranged from 0.638 to 0.872), supporting distinctness yet relatedness of the constructs (see Table 4).

**Table 4:** Results of CFA

Item		Standard Factor Loading	
Recovery-Promoting Competency			
RPC1: Help clients recognize their strengths.		0.708	
RPC5: Help clients not to feel ashamed about their psychiatric condition.		0.757	
RPC7: Help clients find meaning in living with a psychiatric condition.		0.638	
RPC9: Encourage clients to take chances and try things.		0.620	
RPC10: Remind clients of their achievements.		0.644	
RPC11: Help clients feel good about themselves.		0.684	
RPC12: Help clients learn from challenging experiences.		0.701	
RPC13: Help your clients feel hopeful about the future.		0.662	
RPC14: Help clients build their self-confidence.		0.623	
RPC15: Help clients to develop ways to live with their psychiatric condition.		0.637	
RPC16: Help clients understand the nature of their psychiatric condition.		0.647	
RPC17: Demonstrate that you trust your clients.		0.653	
RPC18: Demonstrate that you accept clients’ down times.		0.703	
RPC19: Demonstrate that you understand your clients.		0.704	
RPC20: Demonstrate that you really listen to what your clients have to say.		0.644	
RPC21: Demonstrate that you care about your clients.		0.661	
RPC22: Demonstrate that you treat your clients with respect.		0.685	
RPC23: Demonstrate that you see your clients as people and not just a diagnosis.		0.752	
RPC24: Demonstrate that you believe in your clients.		0.766	
Cultural Humility			
CH1: I want to learn more about people from other cultural backgrounds.		0.737	
CH2: Learning about other cultural backgrounds is an important step in communicating effectively.		0.661	
CH3: People from different countries and cultures have perspectives that I can benefit from.		0.771	
CH5: I make an effort to honor the beliefs, customs, and values of other cultures.		0.674	
CH7: Learning about other cultures enhances a person’s communication skills.		0.664	
CH9: I try to keep an open mind about other cultural norms, beliefs, and values.		0.750	
Recovery Attitudes			
RA2: Recovery can occur even if symptoms of mental illness are present.		0.729	
RA3: Recovering from mental illness is possible no matter what you think may cause it.		0.604	
RA4: All people with serious mental illness can strive for recovery.		0.687	
RA5: People in recovery sometimes have setbacks.		0.753	
RA6: Stigma associated with mental illness can slow down the recovery process.		0.684	
RA7: People differ in the way recover from a mental illness.		0.760	
Model Fit			
χ2(df)	616.461 (df = 431)		
CFI	. 918		
RMSEA [90% CI]	0.056 [0.045, 0.065]		
SRMR	0.054		

Robust χ² (Yuan-Bentler correction); CFI, RMSEA, SRMR are robust estimates

### Structural Equation Modeling

**Fig. 1:**
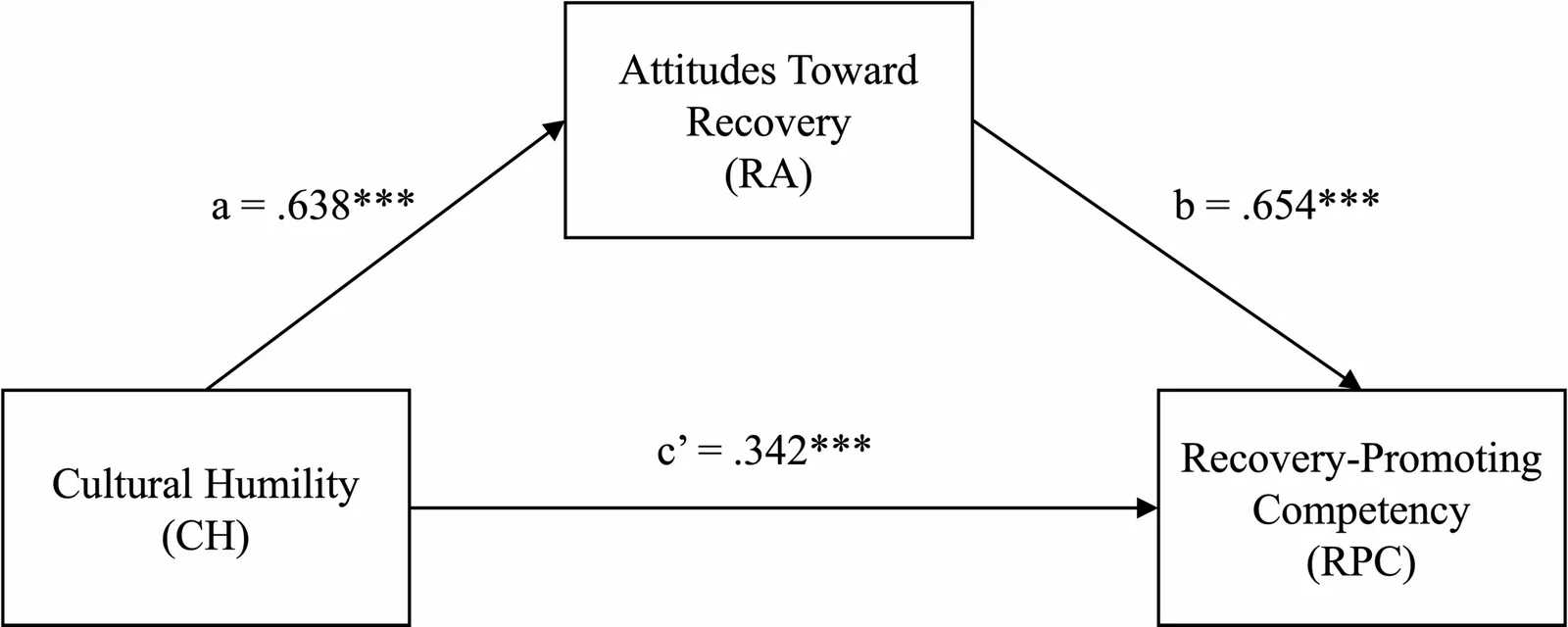
Structural Equation Model. All standardized coefficients. *** *p* < .001. Indirect effect (a × b) = 0.417*** [95% CI: 0.310, 0.670]

Unadjusted mediation model demonstrated acceptable fit to the data (CFI = 0.918, TLI = 0.912, RMSEA = 0.056 [90% CI: 0.045, 0.065], SRMR = 0.054). As hypothesized, cultural humility significantly predicted recovery attitudes (β = 0.638, *p* < .001), which in turn significantly predicted recovery-promoting competencies (β = 0.654, *p* < .001). Importantly, the indirect effect of cultural humility on recovery-promoting competencies through recovery attitudes was significant (β = 0.417, 95% BCa CI [0.310, 0.670], *p* < .001), indicating 54.9% of the total effect was mediated through recovery attitudes. Because the direct effect of cultural humility on recovery-promoting competencies (β = 0.342, *p* < .001) remained statistically significant alongside the significant indirect effect, these results reflect partial mediation (see Fig. 1).

When demographic and professional covariates (age, gender, education, race, years working, and caseload) were included, the mediation pathway remained statistically significant (β = 0.454, 95% BCa CI [0.336, 0.698], *p* < .001), with 60.0% of the effect mediated, indicating that the relationship is robust and not attributable to differences in these characteristics. Modification indices were examined, but no theoretically meaningful adjustments were identified (highest MI = 18.96). Therefore, the model was retained as originally specified (see Table 5).

**Table 5:** Effect Decomposition Table

Pathway	Model 1: Unadjusted (*N* = 188)	Model 2: Adjusted (*N* = 188)
	β [95% CI]	β [95% CI]
**Direct Effects**		
CH → RA (a)	0.638 [0.429, 0.743]	0.650 [0.465, 0.768]
RA → RPC (b)	0.654 [0.608, 1.069]	0.698 [0.607, 1.056]
CH → RPC (c’)	0.342 [0.220, 0.539]	0.302 [0.169, 0.510]
**Indirect Effects**		
CH → RA → RPC	0.417 [0.310, 0.670]***	0.454 [0.336 0.698]***
**Total Effects**		
CH → RPC (direct + indirect)	0.759	0.7516
**Mediation %**	54.9%	60.0%
**Model Fit**	CFI = 0.918, RMSEA = 0.056	CFI = 0.896, RMSEA = 0.054

*p < .001; 95% CI via bias-corrected bootstrap with 5,000 resamples

## Discussion

This study examined the relationship between cultural humility and recovery-promoting competencies among mental health practitioners working with individuals with SMI, and whether recovery attitudes mediate this relationship. Consistent with hypotheses, the direct effect of cultural humility on recovery-promoting competencies was statistically significant, indicating practitioners with higher cultural humility demonstrate higher recovery-promoting competencies. Also, cultural humility was significantly related to recovery attitudes, which were in turn significantly associated with recovery-promoting competencies.

The significant direct effect of cultural humility on recovery-promoting competencies reflects a fundamental conceptual and philosophical alignment between these constructs. Cultural humility emphasizes ongoing openness, self-awareness, egolessness, supportive interactions, and continuous self-reflection (Foronda et al., 2016), whereas recovery-promoting competencies encompass person-centered care, empowerment, holistic well-being, self-reflexive practice, social justice advocacy, and support for meaningful employment (Na et al., 2025). Although these frameworks originate from distinct literatures, they converge on core commitments to equitable partnerships rather than expert-driven hierarchies, recognition of service users as experts in their own lives and recovery processes, support for autonomy and self-determination, and attention to cultural and structural contexts shaping individual experiences. When practitioners engage in ongoing self-reflection regarding their cultural identities, biases, and positions of power, which constitutes the central practice of cultural humility, they are well positioned to enact the relational and ethical orientations that define recovery-promoting competency.

This conceptual convergence is further articulated by Tham and Solomon (2024), who demonstrate that cultural humility and recovery-oriented practice operationalize shared values across five domains: dignity and respect, collaboration and partnership, recognition of individuality, person-centered care, and support for autonomy. Together, this framework suggests that cultural humility functions as a foundational process through which recovery-promoting competencies are expressed in routine practice. Importantly, this alignment reflects more than parallel constructs; rather, the reflective and relational practice of cultural humility directly operationalizes the interpersonal and ethical competencies that define recovery-promoting practice. Through self-reflection, power awareness, and commitment to equitable partnership, practitioners simultaneously enact respect, trust, openness, and genuine connection that constitute recovery-promoting competencies.

The significant indirect effect identifies a mediating mechanism through which cultural humility influences recovery-promoting competencies. This pathway offers empirical support for TRA (Fishbein & Ajzen, 1975), which proposes that behavioral intentions stem from attitudes, and attitudes are shaped by how individuals evaluate potential outcomes and the value they place on those outcomes. Specifically, cultural humility, represented as behavioral beliefs and their evaluations about power, partnership, and service user expertise, shapes practitioners’ recovery attitudes, which in turn motivate behavioral intention to enact recovery-promoting competencies. Through the practice of cultural humility, mental health providers critically reflect on how their social identities and cultural backgrounds influence practitioner–client relationships and actively challenge power dynamics that may marginalize or disempower service users (Tham & Solomon, 2025). This reflective and relational stance fosters the development of recovery-oriented beliefs and attitudes, including the view that recovery is multidimensional, possibly despite ongoing symptoms, and deserving of genuine professional support. These recovery attitudes function as a motivational mechanism that translates practitioners’ power-aware, reflective orientations into behavioral intention to enact recovery-promoting competencies during clinical encounters. As a result, practitioners are more likely to engage service users as active and empowered participants in the therapeutic relationship rather than passive recipients of care, reinforcing the core principles of recovery-oriented practice (Tham & Solomon, 2025).

Lastly, the robustness of findings across unadjusted and adjusted models is noteworthy. Even when controlling for factors that might plausibly influence practitioner competencies such as age, gender, education, race, years of working experience, and caseload size, the mediation pathway remained substantial and statistically significant. This consistency suggests that the hypothesized relationship is not spurious and reflects a genuine underlying process rather than demographic artifact.

Comprehensively, these findings extend prior conceptual and qualitative work on cultural humility by providing empirical evidence that cultural humility is associated with recovery-promoting competencies in mental health practice. Whereas recent work has primarily focused on defining cultural humility or exploring its enactment qualitatively (Bauer et al., 2025; Mosher et al., 2017; Villani et al., 2024), the present study demonstrates the extent to which cultural humility translates into measurable recovery-oriented practice outcomes. Importantly, this study advances the literature by identifying recovery attitudes as a key mediating mechanism linking cultural humility to recovery-promoting competencies. Although recovery attitudes have been recognized as influential in the implementation of recovery-oriented practices (Bedregal et al., 2006; Chang et al., 2025; Mak et al., 2018; Salyers et al., 2013), their role as a mechanism through which cultural humility shapes practitioner behavior has not previously been empirically examined. This study addresses this gap by demonstrating the extent to which cultural humility is associated with recovery-promoting competencies in routine mental health practice.

### Implications for Practice

Practitioners play a critical role in promoting client recovery, highlighting the need for attention to how cultural humility is cultivated within the professional contexts where practitioners are trained and supported. This concern is especially salient given evidence that mental health providers can hold stigmatizing attitudes toward individuals with SMI and that clinical training itself may inadvertently reinforce these attitudes (O’Connor & Yanos, 2021, 2023). Cultivating cultural humility, with its emphasis on self-reflection, power awareness, and recognition of service users as experts in their own lives, offers one concrete orientation through which training and supervision may counteract stigmatizing tendencies and strengthen recovery-oriented engagement. While this paper emphasizes the importance of practitioners engaging in cultural humility, it is important to note that this stance is not developed in isolation. Cultural humility is learned and reinforced through relationships, especially within clinical supervision.

At the same time, cultural humility and recovery attitudes, though conceptually aligned, remain distinct practitioner orientations. These study’s findings indicate that more than half of cultural humility’s effect on recovery-promoting competencies operates indirectly through recovery attitudes, underscoring that these two orientations function as complementary rather than redundant pathways. Training programs should therefore cultivate both in tandem rather than assume that strengthening one will reliably foster the other, ensuring that respect for cultural background and belief in recovery are developed as mutually reinforcing commitments.

Efforts to promote cultural humility should go beyond didactic training for individual practitioners. Instead, cultural humility must also be cultivated within supervisory relationships. Supervision has a central place where values and ways of engaging are made clear, both openly and subtly. The supervisory-practitioner relationship is a key, but underexamined context for enhancing cultural humility. Supervision often involves evaluation and performance monitoring, which can increase power imbalances. If these hierarchies remain unexamined, they may reinforce rigid power structures, limiting practitioners’ capacity for reflection, reflexivity, and critical examination of their own positionality. Without supervision grounded in cultural humility, practitioners may have limited exposure to what occurs in practice, yet may still be expected to provide culturally humble care. This disconnect can undermine the development of authentic practice.

Integrating cultural humility into the supervisory context underscores the importance of modeling this stance within supervision. When supervisors demonstrate openness, ongoing self-reflection, and acknowledgment of their own positionality and limitations, they provide practitioners with firsthand experience of self-awareness and openness central to cultural humility. Supervision in which power is acknowledged and examined allows practitioners to internalize these relational dynamics and apply them to their work with clients.

Moreover, supervisors who embody cultural humility signal that this stance is essential, not optional, for effective clinical practice. Such modeling increases the likelihood that practitioners will carry similar attitudes into their work with clients, particularly when navigating power, culture, and autonomy. In this way, supervision functions as an example of client-practitioner relationship, reinforcing practicing culturally humility through lived relational experience rather than instruction alone. Experiential learning within supervision may therefore be more impactful than purely didactic approaches that describe cultural humility without demonstrating its enactment.

### Organizational Policy Implications

At the organizational level, attention must be directed toward preparing supervisors and providing organizational support for culturally humble supervision. Clinical training programs and mental health organizations should invest not only in practitioner development but also in equipping supervisors with skills, frameworks, and protected time necessary to facilitate discussions about power, culture, and systemic oppression. Structuring supervision to normalize discussions about power, collaboration, and partnership, rather than avoiding them, can enhance practitioners’ awareness of how power dynamics operate, often implicitly, within mental health systems.

Organizations are encouraged to reconceptualize the purpose of clinical supervision beyond administrative oversight and skill monitoring. Supervision that prioritizes cultural humility can strengthen practitioners’ capacity to build equitable, collaborative therapeutic alliances. By embedding cultural humility within supervisory and organizational practices, both practitioners and mental health systems can be better positioned to support recovery for individuals with SMI.

### Limitations and Future Directions

Several limitations should be noted. First, the cross-sectional design and reliance on self-report measures limit causal interpretation and may introduce shared method bias. Although social desirability screening indicated generally acceptable response patterns, practitioners’ self-reported recovery-promoting competencies may not fully reflect their actual clinical behaviors. Additionally, the study’s cross-sectional nature prevents establishment of temporal or causal precedence. While hypothesized mediation model is theoretically sound and consistent with TRA, it is possible that engagement in recovery-promoting competencies shapes recovery attitudes, or that unmeasured third variables influence the observed associations. Future studies could address these limitations by incorporating longitudinal designs, behavioral observations, or multi-source assessments including client-reported outcomes and direct observational data collected during clinical encounters. Such designs would enable stronger causal inference and clarify the temporal dynamics of these relationships.

Second, this study employed non-probability snowball and convenience sampling strategies. While the sample included practitioners from diverse professional backgrounds, practice settings, and geographic regions across the United States, this sampling approach may have preferentially recruited individuals already engaged in or interested in recovery-oriented and culturally responsive practice, potentially inflating effect estimates. Future research using probability-based or stratified sampling strategies would strengthen external validity and allow for more defensible generalizations to broader practitioner populations.

Third, our research question, analytic framing, and eligibility criteria were intentionally scoped to practitioners working with individuals with SMI (requiring at least 40% SMI caseload), and this scope carries interpretive implications. The strength of association between cultural humility and recovery attitudes observed here may reflect integration that develops through sustained SMI-focused practice. Among clinicians whose primary work lies outside SMI contexts, these constructs may be more loosely coupled, with respect for cultural background and belief in recovery experienced as distinct rather than mutually reinforcing. Future research should examine whether the mediation pathway observed here extends to broader practitioner populations, and whether the coupling between cultural humility and recovery attitudes varies with SMI practice intensity, clarifying whether integration emerges organically through SMI-focused experience or requires deliberate cultivation through training.

Fourth, measurement considerations warrant attention. Cultural Humility Self-Assessment Scale (Whatley et al., 2024) emphasizes openness and learning about diverse cultural backgrounds, capturing an important dimension of cultural humility. However, it may not fully operationalize the relational and power-aware dimensions central to cultural humility as originally conceptualized by Tervalon and Murray-García (1998). Specifically, it may not capture practitioners’ critical awareness of their own power, privilege, and positionality within provider–service user relationships, or their efforts to redress power imbalances through partnership. This measurement gap reflects a broader field-level challenge, as to our knowledge no existing instrument comprehensively captures all core dimensions of cultural humility as a distinct construct separate from cultural competence. Continued measurement development and validation are needed to advance empirical research examining cultural humility’s role in clinical practice outcomes.

Similarly, Recovery Attitudes Questionnaire (Borkin et al., 2000), while widely used, has documented limitations regarding psychometric properties and dimensional structure (Jaeger et al., 2013). Additionally, evidence suggests RAQ may not function equivalently across cultural contexts, with validation challenges reported in non-U.S. samples (Daguman & Taylor, 2024; Hungerford, 2014; Wilrycx et al., 2012). Future research would benefit from developing or utilizing more robust and culturally sensitive measures of recovery attitudes that capture the full complexity of how practitioners’ beliefs about recovery translate into practice. More broadly across the three measures, several items were dropped from each measure based on the 0.60 EFA loading threshold (3 from CHS, 1 from RAQ–7, 5 from RPC). While this improved psychometric coherence, it also implies that our measures captured somewhat narrower content than the original instruments, which should be considered when interpreting findings and replicating this work.

## Conclusion

Findings provide empirical evidence for a theoretically grounded pathway through which cultural humility shapes recovery-promoting competencies among mental health practitioners. By applying TRA, this study clarified that cultural humility operates through the development of recovery attitudes, which in turn motivate practitioners’ behavioral intention to engage in recovery-promoting practice. Results suggest that practitioners’ reflective, power-aware orientations grounded in cultural humility fundamentally shape attitudes necessary to authentically enact recovery-promoting behaviors in clinical practice. As mental health systems continue to prioritize recovery-oriented care, investing in practitioners’ cultural humility development may be a critical strategy for ensuring that recovery values are enacted with genuine commitment and relational authenticity.

## Data Availability

The datasets generated and analyzed during this study are not publicly available due to confidentiality and privacy protections for research participants. The data were collected via anonymous Qualtrics survey with participant consent limited to use by the research team. Access to the data may be requested from the corresponding author (S.N., subinna@upenn.edu) subject to approval by the University of Pennsylvania Institutional Review Board and data use agreements that protect participant confidentiality.
